# Inflammatory burden in dialysis patients: the role of alpha defensin

**DOI:** 10.3389/fimmu.2025.1718452

**Published:** 2026-01-14

**Authors:** Maanit Shapira, Adib Abo Aqil, Ameena Zahalka, Isis Abumouch, Naama Amsalem, Rami Abu Fanne

**Affiliations:** 1 Division of Laboratories, Hillel Yaffe Medical Center, Hadera, Israel; 2 Cardiology Department, Hillel Yaffe Medical Center, Hadera, Israel, and the Ruth and Bruce Rappaport Faculty of Medicine, Technion, Israel; 3 Nephrology Department, Hillel Yaffe Medical Center, Hadera, Israel

**Keywords:** alpha-defensin, CRP, dialysis, inflammation, lymphocytes, neutrophils

## Abstract

**Introduction:**

The major neutrophilic peptide alpha-defensin plays a pivotal role in atherogenesis. Atherosclerosis is more frequent in dialysis patients, increasingly ascribed to chronic low-grade inflammation. We investigated the potential association between dialysis treatment and circulating alpha-defensin levels.

**Methods:**

In a cohort of hemodialysis (HD) patients, plasma alpha-defensin concentrations were determined immediately before and after a dialysis session. Blood samples were also tested for CBC, CRP, lipid profile, and troponin levels. Body weight change, Urea Reduction Ratio and Kt/V were used to assess dialysis adequacy. Patients were divided into two groups based on alpha-defensin increase post dialysis. Groups were compared for dialysis adequacy, CBC, CRP, LDL levels, and the incidence of new documented coronary artery narrowing post HD initiation. The study was approved by the local IRB and all patients were consented.

**Results:**

A total of 37 HD patients (55% males, median age 66.5 (60.3–78 years)) were recruited. There was a marked surge in median alpha-defensin levels after HD [11,571 *vs*. 16,661 ng/ml, p=0.009]. Overall, alpha-defensin levels increased in 65% of cases, whereas CRP levels showed no significant rise following dialysis. Similarly, platelet and neutrophil counts exhibited no significant change. Kt/V values were found favorable in HD patients with alpha-defensin decrease (1.48 *vs*. 1.37, P = 0.24), corresponding to a higher body weight decrease post dialysis (2.4% *vs*. 1.75%). Moreover, the HD group with alpha-defensin increase was more prone to sustain new cardiovascular events (12.5% *vs*. 0% at a median time of 5 (3.75-6.57) years), despite demonstrating a better blood lipid profile (LDL 63 *vs*. 87 mg/dl).

**Conclusion:**

HD is an alpha-defensin generating procedure. Patients are potentially predisposed to atherosclerosis because of their enhanced alpha defensin secretion. alpha-defensin might evolve as a potential therapeutic target for atherosclerosis mitigation in this high-risk population. However, this remains to be validated in future research.

## Background

Chronic kidney disease (CKD), and particularly end-stage renal disease (ESRD), is a well-established risk factor for cardiovascular disease. Despite extensive research, the precise mechanisms linking renal failure, especially dialysis patients, to accelerated atherosclerosis remain incompletely defined. A leading paradigm implicates the active, low-grade chronic inflammatory status induced by dialysis as a key driver for atherogenesis (1, 2). The role of inflammation as a significant cardiovascular risk factor in dialysis patients is further pointed by the limited efficacy of established preventive pharmacotherapies (such as aspirin and statins which significantly reduce cardiovascular events in the general population) to confer similar benefit in dialysis patients (3, 4).

Elevated levels of inflammatory markers such as C-reactive protein (CRP) and pro-inflammatory cytokines are frequently observed in ESRD. Among these, interleukin-6 (IL-6) has emerged as a robust prognostic marker (5). Observational data further suggests superior early survival in PD patients, partially attributed to lower systemic IL-6 levels and comparatively lower CRP concentrations (6). However, given the non-specificity of CRP as a biomarker, attention has shifted toward more mechanistically relevant inflammatory mediators.

Alpha-defensin (also known as human neutrophil peptides 1–3, or HNP1–3) has recently emerged as a promising candidate. These 4-kDa cationic peptides are abundantly expressed in neutrophil azurophilic granules, constituting approximately 5% of the total neutrophil proteins. Structurally, they are characterized by a conserved triple β-strand conformation stabilized by three disulfide bridges (7). Beyond their antimicrobial properties, alpha-defensins play multifaceted roles in immune regulation and vascular pathology. Growing evidence implicates alpha-defensins as active participants in atherogenesis and thrombosis. These peptides enhance platelet activation (8), impede LDL and Lp(a) catabolism by vascular cells (9), promote lipid retention within the extracellular matrix (10), and disrupt endothelial function (11, 12) and tPA-mediated fibrinolysis (13). Histological analyses have identified alpha-defensin deposits in the intima and media of human carotid and coronary arteries affected by atherosclerosis, reinforcing their pathogenic role (14, 15).

Our group was the first to demonstrate a causal role of alpha-defensin in atherosclerosis using a transgenic mouse model (16). We showed that alpha-defensin forms stable complexes with LDL, altering its composition and promoting vascular deposition—even under normolipidemic conditions. Additionally, colchicine therapy was shown to counteract this phenotype by stabilizing neutrophils and suppressing alpha-defensin release. More recently, we identified potent pro-thrombotic effects of alpha-defensin, including accelerated thrombus formation and altered clot architecture (17); the resulting fibrin networks are denser, more compact, and resistant to fibrinolysis, promoting sustained *in vivo* thrombosis.

Clinically, elevated alpha-defensin levels have been associated with adverse cardiovascular outcomes, including acute myocardial infarction (18), cardiovascular death in patients with peripheral artery disease (19), and decompensated heart failure (20). Collectively, these findings position alpha-defensin as a biologically plausible, measurable, and potentially modifiable mediator linking neutrophil-driven inflammation with both atherogenesis and atherothrombosis. A previous study by Grupp et al. (21) found comparable serum alpha-defensin levels in hemodialysis patients and in healthy volunteers with similar dynamics post infectious insults. Nonetheless, serum α-defensin levels are artificially elevated by ex vivo neutrophil degranulation during coagulation, obscuring dynamic changes, whereas plasma measurements more accurately reflect *in vivo* circulating levels (17). In this study, we tested plasma alpha-defensin levels aiming to determine whether dialysis itself serves as a stimulus for alpha-defensin secretion.

## Methods

### Study population

This was a prospective non-randomized study carried out at Hillel Yaffe Medical center (Hadera, Israel). Over a period of 9 months from February to October 2020, all consecutive hemodialysis patients aged ≥ 18 years were prospectively included in the study. We excluded patients on antibiotic treatment at admission, inflammatory/infectious disease in the last 3 weeks, chronic immunodeficient status, known cancer, chronic anti-inflammatory medication, and chronic inflammatory disease. The study was conducted in accordance with the Declaration of Helsinki and approved by the HYMC Institutional Review Board (protocol code is 123-18-HYMC).

### Demographic variables documentation

Demographic variables were recorded from patients’ files. Body weights were measured before and at the end of each dialysis session. The effectiveness of dialysis was evaluated using a 3-month mean of Urea Reduction Ratio- URR measurements: one month before enrollment, the month of enrollment, and the month thereafter, providing a stable estimate of each patient’s baseline adequacy. In addition, the KTV value was calculated on the day of dialysis. In addition, we conducted long-term follow-up to assess the incidence of new cardiovascular events.

### Alpha-defensin measurement

Serial alpha-defensin measurements were obtained during a single prespecified hemodialysis session through dialysis access: paired serum and plasma samples immediately before dialysis, followed by serial plasma sampling at one hour, two hours after dialysis initiation, and at the end of the dialysis session. This approach was used to minimize analytical variability and ensure that the observed post-dialysis dynamics reflect true biological responses rather than assay noise. All plasma samples were processed using a standardized pre-analytical protocol applied uniformly across participants. Venous blood was collected into K2-EDTA tubes and processed promptly within 30 minutes of phlebotomy. Samples were centrifuged at 4°C (1,500 × g for 10 minutes). Plasma was carefully aspirated without disturbing the buffy coat and subjected to a second clarification centrifugation at 4°C (10,000 × g for 5 minutes) to obtain platelet-poor plasma. Aliquots were transferred into polypropylene tubes and stored at −80°C until analysis. All samples were thawed only once prior to analysis and alpha-defensin levels were measured within a period not exceeding 3 months. Plasma alpha-defensin levels (HNP1-3; HyCult Biotechnology, Kit#-HK317) were measured using sandwich enzyme-linked immunosorbent assay (ELISA) kits according to the manufacturers’ protocols. Alpha-defensin concentrations were calculated from the Alpha-defensin standard curves. The lower limit of detection was 156 pg/mL. Additionally, 0.5 hour before dialysis blood samples were tested for blood count, liver enzymes, thyroid function, HbA1c, C-reactive protein, troponin and creatine phosphor kinase. At the end of dialysis blood samples were tested for blood count and CRP levels.

### Statistical analysis

Statistical analyses were performed using SPSS version 28.0. Continuous variables were tested for normality (Kolmogorov–Smirnov) and are presented as median (IQR). Between-group comparisons were conducted using the Mann–Whitney U test for continuous variables and Chi-square or Fisher’s exact test for categorical variables. Paired pre- and post-dialysis comparisons and repeated intradialytic measurements were analyzed using the Wilcoxon signed-rank test. Cardiovascular event rates were compared using Fisher’s exact test. All tests were two-tailed, and p < 0.05 was considered statistically significant; analyses of long-term outcomes were exploratory.

## Results

A total of 37 hemodialysis patients were recruited, 50% males, median age (IQR1-3) 68.5 (61.3-78) years. The characteristics of the patients are presented in **Table 1**. Intradialytic plasma alpha-defensin levels demonstrated high internal consistency across the three post-initiation time points, and therefore the end-of-dialysis plasma value was selected as the representative post-dialysis measurement for all analyses.

**Table 1:** Baseline patients characteristics.

Variable	Decrease group (n=13)	Increase group (n=24)	p-value
Years of Dialysis	7.0 ± 6.8	4.4 ± 2.4	0.2
Kt/V	1.5 ± 0.2	1.4 ± 0.2	0.23
Age (years)	65.1 ± 12.8	65.0 ± 12.9	0.99
FX dialyzers	92%	79%	0.39
E190 dialyzers	8%	21%	0.39
Weight Before (kg)	78.2 ± 23.1	84.0 ± 17.1	0.44
Weight After (kg)	76.2 ± 22.8	82.5 ± 16.9	0.44
Alpha-defensin (pg/ml)	18,800 (14,746–23,000)	8,479 (6,657–12,783)	0.003
Average URR	0.71 ± 0.05	0.70 ± 0.06	0.6
PVD (%)	7.7% (1/13)	37.5% (9/24)	0.06
Diabetes (%)	69.2% (9/13)	79.2% (19/24)	0.7
Gender (Female%)	38.5% (5/13)	50.0% (12/24)	0.73
Ethnicity (Arab %)	30.8% (4/13)	41.7% (10/24)	0.72

Values are presented as median (interquartile range) for continuous variables and number (percentage) for categorical variables. PVD, peripheral vascular disease; URR, Urea Reduction Ratio.

**Figure 1:**
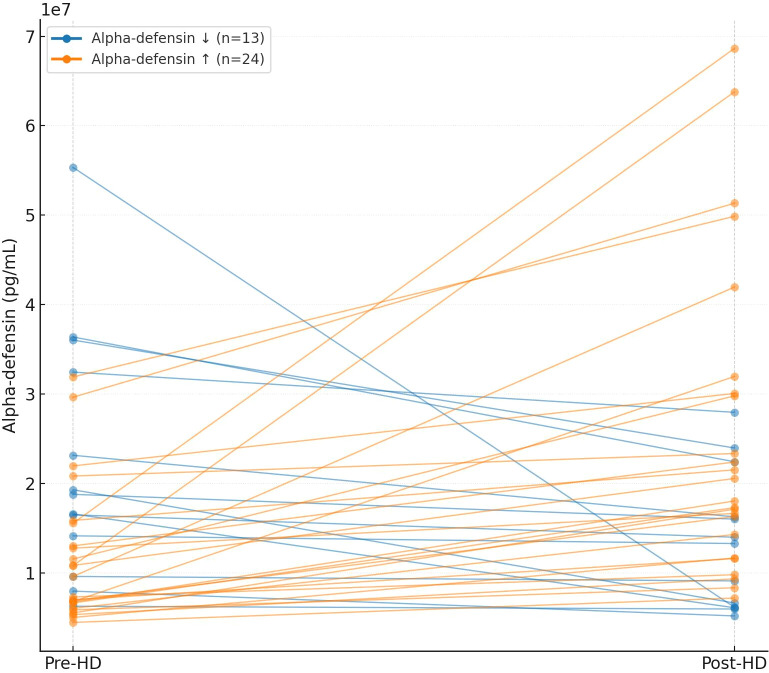
Paired “slope” plot showing pre- *vs* post-HD alpha-defensin for each patient. It also includes medians (IQR) and paired Wilcoxon p-values for the overall cohort and each subgroup.

Overall, 65% of patients exhibited a marked post-dialysis increase in plasma alpha-defensin levels compared to pre-dialysis measurements (**Figure 1**). In the alpha-defensin decrease group, median levels declined from 18,800 pg/mL (IQR: 14,746–23,000) to 13,314 pg/mL (IQR: 7,271–16,221), p=0.0001. Conversely, in the alpha-defensin increase group, levels rose from 8,479 pg/mL (IQR: 6,657–12,783) to 19,310 pg/mL (IQR: 14,320–30,076), p=5.96e-08. We next asked what characterizes the 65% of patients with alpha-defensin increase post dialysis; are those technical parameters related to the dialysis procedure or patient related parameters. Although **Table 1** displays numerically higher KTV (1.5 ± 0.2) *vs*. 1.4 ± 0.2), and relative weight reduction (2.5% *vs*. 1.75%) in patients with alpha-defensin decrease, the differences were not statistically significant. We finally explored the distribution of dialyzer type between groups. In the alpha-defensin decrease group, 92% of dialysis used FX dialyzers and 8% used E190, whereas in the increase group 79% used FX and 21% used E190, p= 0.39.

We then tested the dynamics of different relevant blood indices before and after dialysis in both groups (**Table 2**). Alpha-defensin levels were significantly changed in both groups. On the other hand, CRP levels before and at the end of dialysis showed no significant differences in both groups. The same trend was documented with WBC, neutrophils, lymphocytes, and platelets. We also assessed metabolic variables including lipid profile, diabetes control, and troponin level in both groups (**Table 3**). The prevalence of diabetes was non significantly higher among the subgroup with alpha- defensin increase, 79% *vs*. 69.2%, and the corresponding HbA1c levels were 6.1 (5.5-6.7) *vs*. 5.7 (5.2-6.2), respectively. Considering the lipid profile, 56.5% of the subgroup with alpha-defensin increase were hyperlipidemic compared to 27.3% in patients with alpha-defensin decrease. However, the median LDL values were 63 (38-89) *vs*. 87 (72-100) mg/dl, respectively, p=0.03. As shown, apart from cholesterol levels, no significant differences were documented.

**Table 2:** Different blood indices dynamics before and after dialysis in both groups.

Variable	Alpha-defensin increase			Alpha-defensin decrease		
	Predialysis-	Postdialysis-	P	Predialysis-	Postdialysis-	P
Alpha-defensin (pg/ml)	8,479 (6,657–12,783)	19,310 (14,320–30,076)	5.96e-08	18,800 (14,746–23,000)	13,314 (7,271–16,221)	0.0001
CRP (mg/L)	7.5 (4-15.8)	7.2 (4.2-13)	0.74	6.6 (4.7-14.5)	6.5 (5.7-13.2)	1
WBC (×10^9^/L)	5.76 ± 1.96	5.58 ± 1.61	0.25	6.31 ± 2.83	5.84 ± 2.26	0.43
Platelets (×10^9^/L)	186.7 ± 51.3	196.6 ± 50.6	0.03	195.4 ± 44.7	197.4 ± 41.3	0.92
Lymphocytes (%)	23.8 ± 26.9	17.06 ± 4.50	0.13	22.62 ± 9.23	20.20 ± 8.72	0.32
Neutrophils (×10^9^/L)	4.04 ± 1.56	4.05 ± 1.30	0.87	4.06 ± 2.22	3.93 ± 1.73	0.7

**Table 3:** Key baseline metabolic variable stratified by alpha-defensin trend.

Variable	HD- Alpha-defensin decrease	HD- Alpha-defensin increase	P
Cholesterol (mg/dL)	173.5 ± 47.2	134.8 ± 38.5	0.01
Triglycerides (mg/dL)	205.9 ± 168.6	138.2 ± 83.7	0.38
HDL (mg/dL)	38.4 ± 9.7	39.1 ± 13.5	0.66
LDL (mg/dL)	93.8 ± 38.1	68.2 ± 28.5	0.03
HbA1c (%)	5.94 ± 0.92	6.45 ± 1.41	0.2
Troponin (ng/l)	72 (52-183)	77 (42-146)	0.56

Variables are presented as Mean ± SD. HDL, high density lipoprotein; LDL, low density lipoprotein; HbA1c, hemoglobin A1c.

### The occurrence of new CV events after dialysis initiation

We assessed the incidence of new cardiovascular events necessitating percutaneous coronary intervention among patients following the initiation of dialysis. During a median follow-up of 5.0 years (IQR, 3.75–6.57), 12.5% of patients in the alpha-defensin–increase group experienced a new cardiovascular event, whereas no such events were observed among patients in whom alpha-defensin levels decreased; however, this difference was not statistically significant (p = 0.54).

## Discussion

Inflammation is intricately linked to renal dysfunction, irrespective of the use of maintenance hemodialysis (HD) (22). Elevated concentrations of inflammatory biomarkers have consistently been associated with increased mortality among dialysis-dependent individuals (23).

The present study demonstrates that hemodialysis is predominantly a neutrophil-activating procedure, as evidenced by a significant post-dialysis rise in plasma alpha-defensin levels. This response was observed despite comparable intradialytic weight reduction and similar dialysis adequacy (URR) between patients exhibiting alpha-defensin increases and decreases, arguing against differential ultrafiltration or hemoconcentration as explanatory mechanisms. Collectively, these findings support the interpretation that alpha-defensin elevation reflects an active, procedure-related innate immune response rather than a passive concentration effect.

Our findings differ from those reported by Grupp et al. (21), who observed no significant intradialytic change in alpha-defensin expression. Interpretation of alpha-defensin measurements is highly dependent on pre-analytical handling. Although Grupp et al. refers to “serum alpha-defensin levels,” its Methods describe plasma-based measurements, suggesting imprecise terminology rather than true serum analysis. Importantly, differences in plasma preparation, including centrifugation speed, number of centrifugation steps, and time from blood collection to processing—can substantially influence measured alpha-defensin concentrations. Delayed or low-speed centrifugation (230 × g for 5 minutes in Grupp study *vs*. 1,500 × g for 10 minutes in our) may permit residual cellular contamination and ex vivo neutrophil activation, leading to artifactual elevation of alpha-defensin levels. Such methodological variability may therefore contribute to heterogeneity and discrepancies across studies.

Importantly, the intradialytic alpha-defensin response occurred in the absence of corresponding changes in CRP, highlighting a dissociation between alpha-defensin dynamics and conventional inflammatory biomarkers. CRP primarily reflects chronic low-grade inflammation and lacks the temporal resolution required to capture acute, procedure-related immune activation (24, 25). This pattern is biologically coherent, as CRP is a hepatic acute-phase reactant whose systemic elevation typically becomes evident only 6–8 hours following an inflammatory insult, while the average HD session spans just 3 to 4 hours (26). These findings underscore the potential value of alpha-defensin as a dynamic biomarker that reflects immediate neutrophil stress during dialysis.

When comparing the two groups, baseline plasma alpha-defensin levels were significantly higher in patients who subsequently exhibited a post-dialysis decrease. However, baseline alpha-defensin values represent a single time-point measurement and may not reliably reflect each patient’s chronic inflammatory state. Alpha-defensin is a highly dynamic neutrophil-derived peptide with substantial short-term biological variability, responding rapidly to transient inflammatory, complement-mediated, and mechanical stimuli. In this context, baseline heterogeneity likely reflects inter-individual variability rather than a stable phenotype or intrinsic cardiovascular risk marker.

Notably, the design of the present study specifically addressed this limitation. By performing repeated plasma measurements within the same dialysis session, we demonstrated high intra-session consistency within individual patients, indicating that despite inherent biological variability, the paired pre-/post-dialysis comparison captures a reliable and reproducible biological signal. Accordingly, the primary objective of this study was to characterize the acute dialysis-related effect on alpha-defensin dynamics, rather than to define the prognostic significance of single baseline measurements. Notably, beyond our internal consistency, dialysis-induced neutrophil activation and degranulation have been shown to be highly reproducible across repeated dialysis sessions in multiple mechanistic studies, including reproducible complement activation, oxidative burst, CD11b upregulation, upregulation of leukocyte intracellular RNA transcripts encoding proinflammatory cytokines, and azurophilic granule release (27–31).

Although dialysis adequacy did not differ between groups, a numerical imbalance in dialyzer type was observed, with greater use of FX membranes in the alpha-defensin decrease group and relatively higher use of E190 membranes in the increase group. While this difference was not statistically significant and does not support causal inference, it raises the possibility that membrane characteristics and blood–membrane bio-interaction may influence neutrophil activation independently of solute clearance efficiency. This observation aligns with a broader mechanistic model in which the net intradialytic change in alpha-defensin reflects the balance between dialytic removal of this small, highly cationic peptide and simultaneous procedure-induced neutrophil degranulation. Patients in whom clearance predominates may exhibit a post-dialysis decrease, whereas those with stronger innate immune activation may demonstrate an increase- providing a coherent explanation for the divergent response patterns observed in this cohort.

During long-term follow-up, cardiovascular events were observed exclusively among patients who exhibited intradialytic alpha-defensin surges; however, the small number of events and lack of statistical significance preclude any causal inference. Moreover, the absence of repeated longitudinal alpha-defensin measurements limits assessment of chronic intra-individual variability. Accordingly, this observation should be regarded as exploratory and hypothesis-generating rather than evidence of a prognostic association.

In summary, the present study identifies plasma alpha-defensin as a sensitive marker of acute neutrophil activation during hemodialysis, capturing biological responses that are not reflected by traditional inflammatory markers. These findings highlight the dynamic nature of dialysis-induced innate immune activation and support further investigation of alpha-defensin as a mechanistic and potentially clinically informative biomarker. Prospective longitudinal studies incorporating repeated measurements across multiple dialysis sessions and stratification by membrane type will be required to determine reproducibility and define any prognostic implications for cardiovascular outcomes in ESRD.

### Clinical implications

From a clinical perspective, the present findings suggest that alpha-defensin may serve as a dynamic biomarker of dialysis-induced innate immune and thrombo-inflammatory activation, rather than a static marker of background inflammation. Its principal potential utility lies in identifying patients who exhibit exaggerated intradialytic inflammatory responses, which are not adequately captured by conventional markers such as CRP. In future applications, serial intradialytic alpha-defensin profiling could be used to phenotype individual inflammatory responsiveness to dialysis and to guide strategies aimed at reducing procedure-related inflammation. However, given the exploratory nature of the current data and the limited number of outcome events, alpha-defensin cannot yet be recommended for routine clinical risk stratification, and its role as a clinically actionable biomarker will require validation in large prospective longitudinal studies with repeated sampling and hard cardiovascular endpoints.

### Study limitations

Several limitation must be acknowledged in our study. First, the number of patients recruited is relatively small. Yet, this is the first study to address this new evolving biomarker in dialysis patients. Second, the study was observational and so no causative association between alpha-defensin and CVS events could be drawn. Third, no interventional measures were applied in an attempt to ameliorate alpha-defensin surge including aggravating ultrafiltration or administration of neutrophil stabilizing agent like colchicine. This ought to be addressed in future prospective studies. Lastly, no repeated measurements for alpha-defensin levels across different dialysis sessions were performed; accordingly, the present study characterizes intradialytic alpha-defensin dynamics during a single hemodialysis exposure, and this represents a methodological limitation that is explicitly acknowledged.

## Data Availability

The raw data supporting the conclusions of this article will be made available by the authors, without undue reservation.
